# Impact of SARS-CoV-2 Gamma lineage introduction and COVID-19 vaccination on the epidemiological landscape of a Brazilian city

**DOI:** 10.1038/s43856-022-00108-5

**Published:** 2022-04-13

**Authors:** Cecília Artico Banho, Lívia Sacchetto, Guilherme Rodrigues Fernandes Campos, Cíntia Bittar, Fábio Sossai Possebon, Leila Sabrina Ullmann, Beatriz de Carvalho Marques, Gislaine Ceslestino Dutra da Silva, Marília Mazzi Moraes, Maisa Carla Pereira Parra, Andreia Francesli Negri, Ana Carolina Boldrin, Michela Dias Barcelos, Thayza M. I. L. dos Santos, Bruno H. G. A. Milhim, Leonardo Cecílio Rocha, Fernanda Simões Dourado, Andresa Lopes dos Santos, Victoria Bernardi Ciconi, Caio Patuto, Alice Freitas Versiani, Rafael Alves da Silva, Edoardo Estevam de Oliveira Lobl, Victor Miranda Hernandes, Nathalia Zini, Carolina Colombelli Pacca, Cássia Fernanda Estofolete, Helena Lage Ferreira, Paula Rahal, João Pessoa Araújo, Jamie A. Cohen, Cliff C. Kerr, Benjamin M. Althouse, Nikos Vasilakis, Mauricio Lacerda Nogueira

**Affiliations:** 1 Laboratório de Pesquisas em Virologia, Departamento de Doenças Dermatológicas, Infecciosas e Parasitárias, Faculdade de Medicina de São José do Rio Preto, São José do Rio Preto, São Paulo Brazil; 2 Laboratório de Estudos Genômicos, Departamento de Biologia, Instituto de Biociências, Letras e Ciências Exatas, Universidade Estadual Paulista, São José do Rio Preto, São Paulo Brazil; 3 Instituto de Biotecnologia, Universidade Estadual Paulista, Botucatu, São Paulo Brazil; 4 Departamento de Vigilância Epidemiológica, São José do Rio Preto, São Paulo Brazil; 5 Faculdade Ceres (FACERES), São José do Rio Preto, São Paulo Brazil; 6 Laboratório de Medicina Veterinária Preventiva Aplicada, Departamento de Medicina Veterinária, Faculdade de Zootecnia e Engenharia de Alimentos, Universidade de São Paulo, São Paulo, São Paulo Brazil; 7 Institute for Disease Modeling, Global Health Division, Bill & Melinda Gates Foundation, Seattle, WA USA; 8 University of Washington, Seattle, WA USA; 9 New Mexico State University, Las Cruces, NM USA; 10 Department of Pathology, University of Texas Medical Branch, Galveston, TX USA; 11 Center for Vector-Borne and Zoonotic Diseases, University of Texas Medical Branch, Galveston, TX USA; 12 Center for Biodefense and Emerging Infectious Diseases, University of Texas Medical Branch, Galveston, TX USA; 13 Center for Tropical Diseases, University of Texas Medical Branch, Galveston, TX USA; 14 Institute for Human Infection and Immunity, University of Texas Medical Branch, Galveston, TX USA

**Keywords:** Epidemiology, Viral infection

## Abstract

**Background:**

The emergence of the Brazilian variant of concern, Gamma lineage (P.1), impacted the epidemiological profile of COVID-19 cases due to its higher transmissibility rate and immune evasion ability.

**Methods:**

We sequenced 305 SARS-CoV-2 whole-genomes and performed phylogenetic analyses to identify introduction events and the circulating lineages. Additionally, we use epidemiological data of COVID-19 cases, severe cases, and deaths to measure the impact of vaccination coverage and mortality risk.

**Results:**

Here we show that Gamma introduction in São José do Rio Preto, São Paulo, Brazil, was followed by the displacement of seven circulating SARS-CoV-2 variants and a rapid increase in prevalence two months after its first detection in January 2021. Moreover, Gamma variant is associated with increased mortality risk and severity of COVID-19 cases in younger age groups, which corresponds to the unvaccinated population at the time.

**Conclusions:**

Our findings highlight the beneficial effects of vaccination indicated by a pronounced reduction of severe cases and deaths in immunized individuals, reinforcing the need for rapid and massive vaccination.

## Abstract (web summary)

Banho, Sacchetto et al. perform a genomic epidemiology study of the introduction of the SARS-CoV-2 Gamma variant in a city in Brazil. They show that Gamma displaced seven other circulating variants, led to a rapid increase in COVID-19 prevalence, and to increased risk of severe disease and mortality, especially in the unvaccinated population.

## Plain language summary

SARS-CoV-2, the virus responsible for COVID-19, has evolved into different variants, virus lineages with a slightly different genetic material. Here, we evaluated the introductions of SARS-CoV-2 lineages in a Brazilian city with the third-highest number of confirmed COVID-19 cases in the state of São Paulo. Our analyses revealed several SARS-CoV-2 lineages first detected in the city, but after the introduction of the Gamma lineage, the majority of them were replaced by this variant that became the most common. At the same time, there was an increase in the number of infections, severe disease cases, and deaths, mainly in individuals under 70 years old, the age group that was not fully vaccinated at the time. Our findings further support the evidence that vaccination effectively decreases SARS-CoV-2 spread and transmission.

## Introduction

The severe acute respiratory syndrome coronavirus 2 (SARS-CoV-2) has affected millions of lives worldwide^1^, and with its rapid spread and evolution, variants have emerged and continue to emerge globally^2^. Currently, SARS-CoV-2 variants that present a threat to global health are classified as Variants of Interest (VOIs) and Variants of Concern (VOCs)^3^. To date, all VOCs are descendants of the SARS-CoV-2 strain containing a Spike (S) D614G mutation, which is associated with increased viral fitness by enhancing viral load in the upper respiratory tract favoring viral spread and transmission^4,5^. As of December 2021, five reported lineages had been defined as VOCs, four of them first detected in late 2020: B.1.1.7 (Alpha), first detected in the United Kingdom (UK), has been associated with enhanced transmissibility, higher severity of disease^6,7^, and lower neutralization potential by vaccine and convalescent sera^8^; B.1.351 (Beta), a variant first detected in South Africa, has been associated with an increased risk of transmission and reduced neutralization by monoclonal antibody therapy, convalescent sera, and post-vaccination sera^9–12^; Gamma (P.1), a Brazilian variant derived from the B.1.1.28 lineage, shares some critical mutations in the Spike protein with B.1.351^13^; and B.1.617.2 (Delta), detected in India, has spread and replaced other variants circulating and currently is the dominant lineage globally, and showed significantly reduced neutralizing antibody activity compared with the wild-type strain and other VOCs^14^. The B.1.1.529 variant, first reported to WHO from South Africa on 24 November 2021, was recently advertised as a VOC and named Omicron^15^. It is unclear whether Omicron is more transmissible or causes more severe disease than infections with other variants^15^. However, this decision was based on Omicron’s large number of mutations, especially in the Spike gene (more than 30 mutations)^16^.

The Brazilian VOC, Gamma lineage, was first detected in early December in Manaus, the capital of Amazonas state^17^, and rapidly spread throughout Brazil and to more than 60 countries^2^. Gamma lineage emerged after a period of rapid genetic diversification^17^ and accumulated 17 non-synonymous defining mutations, ten of which are located in the S gene^13,17^, of which K417T:E484K:N501Y were demonstrated to be involved in immune escape^18,19^. Recent studies have reported that Gamma lineage presents an increase in ACE2 receptor affinity due to the presence of the N501Y mutation^18,20^, which considerable impacts its transmissibility rate, shown to be 1.4–2.2-fold higher than the wild type strain^13^. Moreover, Gamma also exhibited a reduction of anti-RBD antibody neutralization^21^, and it has been implicated in breakthrough infections of vaccinated individuals and reinfections^22–24^. Although several studies documented, Gamma’s increased transmissibility^17,25^ and immune evasion^22,24,26–28^, little is known about its association with the severity of COVID-19 disease and mortality risk, which is crucial to better understand and mitigate the severe impact of the ongoing pandemic.

In addition to Gamma lineages emergence and dissemination, Brazil has faced a slow vaccination roll-out, which contributed to prolonging the pandemic and the emergence of additional SARS-CoV-2 variants. The immunization campaign started in February 2021, in descending age order. As of July 26, 2021, 131,946,091 doses of COVID-19 vaccine have been administered, with 44.11% and 17.3% of the population receiving the first and second doses, respectively^29^. Brazil has approved the use of four COVID-19 vaccines: ChAdOx1 nCoV-19 (AZD1222; Oxford-AstraZeneca), CoronaVac (Butantan Institute, São Paulo, Brazil, and Sinovac Life Sciences, Beijing, China), BNT162b2 mRNA (Pfizer/BioNTech), and Ad26.COV2.S (Janssen/Johnson & Johnson), corresponding to 46.4%, 40.8%, 9.9%, and 2.9% of the administered doses^29^, respectively. While most vaccines show high efficacy in preventing severe COVID-19 disease and death^30–35^, the impact of slow vaccination rates on the circulation and spread of VOCs on the epidemiological profiles at the national and local level is still unclear. Here, we report the rapid spread of the Gamma variant following its introduction and dissemination in São José do Rio Preto (SJdRP), Brazil, the municipality with the third-highest number of confirmed COVID-19 cases in São Paulo and where based Hospital de Base (HB), the main responsible for SARS-CoV-2 diagnosis and one of the leading in COVID-19 care and treatment in the state. Moreover, the new lineage introduction drove a clade replacement event, associated with a change in the epidemiological profile, with increased severe COVID-19 cases and deaths, especially in the unvaccinated population.

## Methods

### Study area description

São José do Rio Preto (SJdRP) is in the northeast region of the state of São Paulo (SP), Brazil, with a total of 408,258 inhabitants. One of the largest and most important hospital complexes in the municipality is the Hospital de Base de São José do Rio Preto (HB). The HB is a reference health center serving more than two million inhabitants of the 102 municipalities belonging to the 15th Regional Health Department (RHD XV), headquartered in SJdRP. The HB complex is one of the leading in COVID-19 care and treatment centers in SP state, having the second largest COVID-19 ICU in Brazil, with more than 180 beds and having received more than 5700 admissions so far. Besides, since the beginning of the COVID-19 pandemic, HB is the main health unit responsible for SARS-CoV-2 diagnosis for SJdRP and surrounding municipalities population. The hospital is linked to the Faculdade de Medicina de São José do Rio Preto (FAMERP), an educational facility where Laboratório de Pesquisas em Virologia (LPV) is located and where this research was conducted.

### Moving average analysis

A time-trend analysis was performed using a seven-day moving average of notified cases, severe cases, and deaths related to COVID-19 in SJdRP from March 2020 to May 2021. The data were retrieved from the Public Health System of SJdRP and received from the Reporting Disease Information System (SINAN), using mild respiratory syndrome (e-SUS) and severe acute respiratory syndrome (SRAG) cases databases.

### Samples and molecular investigation

Nasopharyngeal swab samples of residents of SJdRP and nearby cities tested at HB by molecular diagnosis in the COVID-19 routine diagnosis with a positive diagnostic for COVID-19 were obtained between October 2020 and June 2021. The total RNA was extracted from 140 µL of nasopharyngeal swab samples, using QIAamp Viral RNA Mini Kit (QIAGEN, Hilden, Germany), following the manufacturer’s instructions. SARS-CoV-2 RNA investigation was performed by one-step real-time polymerase chain reaction (RT-qPCR) using primers and probes targeting the envelope (E), the nucleocapsid (N) region of the SARS-CoV-2 genome, and the human RNAse P according to GeneFinder COVID-19 Plus RealAmp Kit (OSANG Healthcare, KOR) (the manufacturer does not provide sequences of the primers and probe)^36^. The RT-qPCR was conducted in a QuantStudio 3 Real-Time PCR System (Thermo Fisher Scientific, USA) with the following conditions: 50 °C for 20 min for the reverse transcription, 95 °C for 5 min for denaturation, followed by 45 cycles of denaturation at 95 °C for 15 s, and annealing at 58 °C for 60 s The results were analyzed in QuantStudio 3 software v1.5.1 (Thermo Fisher Scientific, USA) and were interpreted as cycle quantification value (Cq) less or equal 40 as positive and Cq more than 40 as negative. Positive and negative controls, included in the GeneFinder COVID-19 Plus RealAmp Kit (non-infectious DNA plasmids coding for the SARS-CoV-2 E gene and N gene), were used in the assay.

The study was approved by the institutional review board (IRB) of the Ethics Committee of the Faculdade de Medicina de São José do Rio Preto (protocol number: CAE# 31588920.0.0000.5415). Informed consent was not required, given that all data were analyzed anonymously with the total confidentiality of each participant.

### Whole-genome sequencing

Whole-genome sequencing was performed using Next-Generation Sequencing (NGS) technology. The cDNA synthesis, whole-genome amplification, and library preparation were carried out following the instructions provided by Illumina CovidSeq Test (Illumina Inc, USA) and QIAseq SARS-CoV-2 Primer Panel (Qiagen, USA). The quality and size of the libraries were verified by Agilent 4150 TapeStation (Agilent Technologies Inc, USA). Libraries were pooled in equimolar concentrations, and the sequencing was implemented on the Illumina MiSeq System (Illumina Inc, USA), using MiSeq Reagent Kit v2 (read length of 2 × 150 bp) (Illumina Inc, USA).

### Genome assembling and lineage analyses

The quality of FASTQ sequencing data was checked using FastQC software v0.11.9 (http://www.bioinformatics.babraham.ac.uk/projects/fastqc), and trimming was performed in Geneious Prime v. 2021.1 (https://www.geneious.com/), using the plugin BBDuk v. 37.25, to remove primer sequences, adapters, and low-quality bases. A minimum Phred score of Q30^37^ and a minimal read length of 75 base pairs (bp) were used. The cleaned paired-end reads were mapped against the hCoV-19/Wuhan/WIV04/2019 (EPI_ISL_402124) genome, available at EpiCovin GISAID database^38^ (https://www.gisaid.org/), considering a minimum 50 bp overlap, minimum identity of 90% and maximum mismatches of 10% per read^39^. SNPs were identified using default settings and minimum coverage of five reads per site. The genomes were submitted to Pangolin COVID-19 Lineage Assigner Tool v.3.1.14^2^ to confirm the variant classification (https://pangolin.cog-uk.io/).

### Geoprocessing

A database was created with SARS-CoV-2 identified lineages, location/municipality origin, geographic coordinates, and date of the collected sample. Shapefiles of the areas were downloaded from Instituto Brasileiro de Geografia e Estatística (IBGE), and maps were created using the QGIS software version 3.8.2 (http://qgis.org). Satellite images of the study areas were generated using the Google Maps Geocoder v. 0.0.3 plugin implemented in QGIS software version 3.8.2 (http://qgis.org).

### Phylogenetic and evolutionary analyses

The datasets used for the phylogenetic analysis included Brazilian SARS-CoV-2 complete genomes sequences and the reference genome hCoV-19/Wuhan/WIV04/2019 (EPI_ISL_402124) retrieved from the GISAID database (https://www.gisaid.org/) (Supplementary Data 1). The nucleotide sequences were aligned using MAFFT multiple sequence alignment software version 7.271^40^. Time-scale phylogenetic trees using the Maximum-likelihood (ML) method were reconstructed in IQ-TREE v. 2.0.3^41^, using the best-fit model of nucleotide substitution, according to Bayesian Information Criterion (BIC), inferred by ModelFinder application^42^. The reliability of branching patterns was tested using a combination of Ultrafast Bootstrap (UFBoot) and SH-like approximate likelihood ratio test (SH-aLRT)^43^. To investigate the temporal signal from the ML tree, we regressed root-to-tip genetic distances against sample collection dates using TempEst v 1.5.1 (http://tree.bio.ed.ac.uk)^44^.

### Statistics and reproducibility

#### Epidemiologic analyses

Estimation of the effective reproductive number (Reff, the number of secondary infections for each primary infection) for SARS-CoV-2 over the study period was carried out using the package EpiEstim^45^ in R version 4.0.4^46^. Detailed methods can be found in the documentation of the EpiEstim package. We fit the time-varying Reff assuming a parametric serial interval with a mean and standard deviation of 5 days^47^ using a 21-day sliding window. To assess potential shifts in severity, we calculate the percentage of all severe cases for all age groups and those 70 and older. We compare the proportion of deaths, non-severe, and severe cases by age to look for qualitative shifts in age distributions due to changing transmissibility or virulence of the changing variants and any potential vaccine effects. To assess the magnitude of increases in deaths as a function of Gamma prevalence, we fit both Poisson and Negative Binomial (to capture any potential over-dispersion in the numbers of deaths) regression models to calculate incidence rate ratios for death by age, adjusting for the count of tests performed on that day. Negative Binomial regressions were fit using the MASS package in R. We then compare the fits of the two regression methods using the Akaike information criterion (AIC), which captures model fit while balancing model complexity. Finally, we include an index of social mobility^48^ to track time spent away from the cellphone’s predominant nighttime location. The index of social mobility for the main cities of São Paulo State is measured by the *Sistema de Monitoramento Inteligente de São Paulo* (SIMI-SP) through an agreement between the telecommunication companies (*Associação Brasileira de Recursos em Telecomunicações- ABR Telecom*) and the *Instituto de Pesquisa Tecnológicas* (IPT). The index of social mobility is calculated based on georeferencing obtained by the cell phone antennas, which set the time for a smartphone between 10 pm–2 am. If this smartphone had a displacement of more than 200 meters during the day, it would be considered out of social isolation. This index is updated daily and respects the privacy of smartphone users. This monitoring is carried out in 139 cities of the São Paulo State, and the index of social mobility data are available at: https://www.saopaulo.sp.gov.br/coronavirus/isolamento/. The code for these analyses is available on Github^49^.

#### Vaccination efficacy

The relationship between vaccination rate by age and rates of cases, severe cases, and deaths was determined by ordinary least squares fits via the Python library stats models (v0.13.1). For each age group, the proportion of cases, severe cases, and deaths in that age group relative to people aged under 60 (the age cutoff for the vaccine rollout) was calculated (a) for the period up the start of the vaccine rollout for that age group, and (b) for the period starting from two weeks after the end of vaccine rollout for that age group and ending on 29 May 2021. For example, if there were 60% as many deaths in people aged 90+ compared to people aged under 65 before the vaccine rollout began, and 30% as many deaths in people aged 90+ compared to under 65 after the vaccine rollout ended, then the change in the proportion of deaths would be –50%. Coverage was calculated as the number of doses divided by target population size divided by two (since two doses are required for full coverage). An ordinary least squares fit was performed using Python 3.8 (https://www.python.org/), stats models v0.12.2 (https://www.statsmodels.org/stable/index.html) to calculate the slope of best fit between the vaccine coverage and the change in the proportion of each of cases, severe cases, and deaths, with the intercept fixed at zero. The code for these analyses is available on Github^49^.

## Results

### COVID-19 samples characterization

**Fig. 1:**
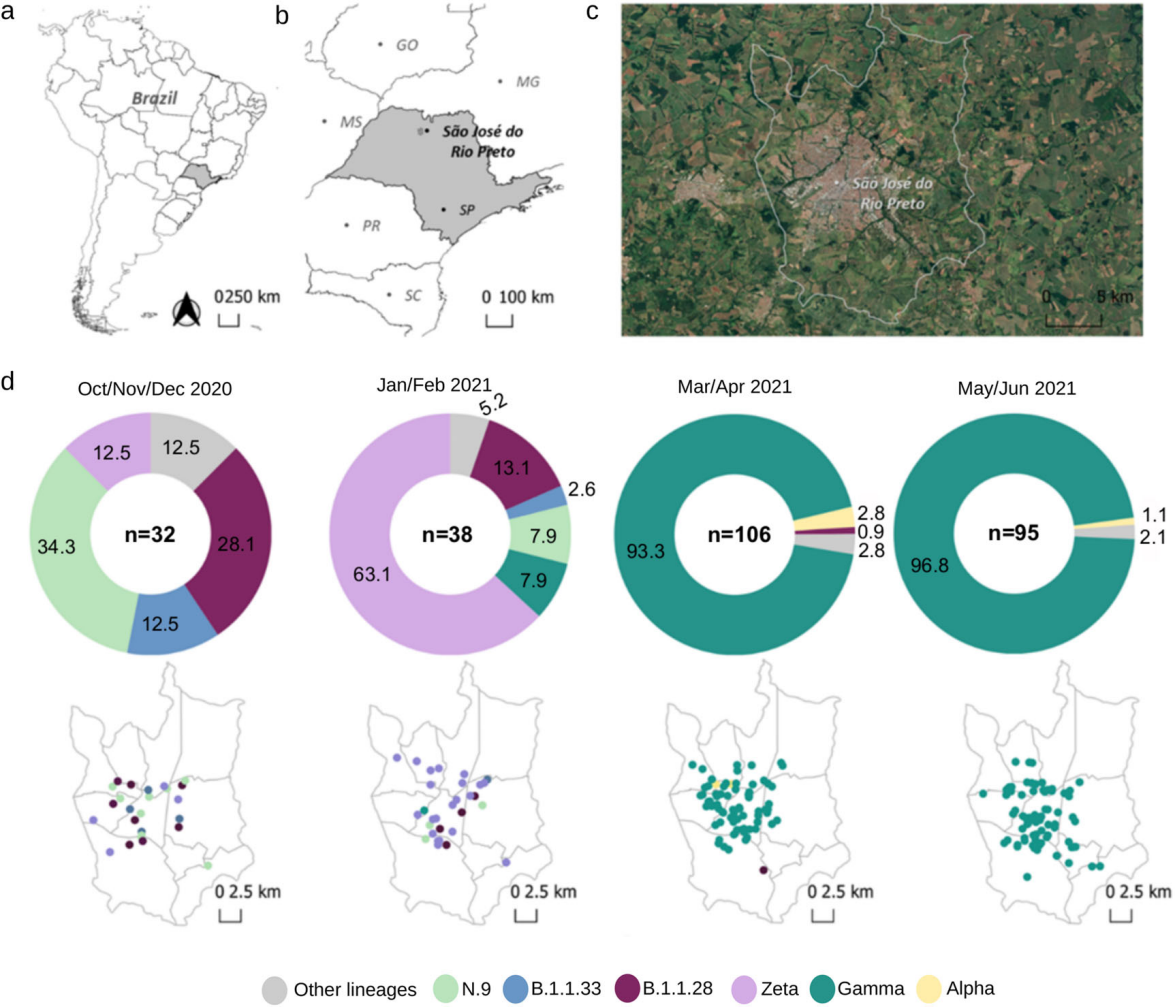
Geographic and temporal distribution of SARS-CoV-2 variants detected in the study area of São José do Rio Preto, São Paulo, Brazil, from October 2020 to June 2021 by genomic surveillance. **a** Geopolitical map of Brazil and São Paulo state highlighted in grey. **b** Map of São Paulo state (in grey) indicating the municipality of São José do Rio Preto (SJdRP), located in the Northwest region. **c** Satellite image of SJdRP generated using the Google Maps Geocoder v. 0.0.3 plugin implemented in QGIS software version 3.8.2 (http://qgis.org). **d** Prevalence, and distribution of SARS-CoV-2 variants detected in SJdRP from October 2020 to June 2021 by genomic surveillance. The information about the detected SARS-CoV-2 variant’s location and collection date was used to create the maps in QGIS software version 3.8.2 (http://qgis.org).

As of December 2, 2021, SJdRP, a medium-sized city in the Northwest region of São Paulo state, Brazil (Fig. 1a–c), has the third-highest number of confirmed COVID-19 cases in the state of São Paulo, reaching 96,151 cases and 2822 deaths, with a case fatality rate of 2.9%^50^. A seven-day moving average of COVID-19 cases showed an increase in hospitalized cases and deaths from February 2021 to April 2021 compared to a year ago (Supplementary Fig. 1). These numbers raised concerns on the epidemiological landscape in SJdRP and the surrounding region. To better understand the events underlying the circulation, transmission, and evolutionary dynamics of SARS-CoV-2, extensive genomic surveillance was implemented since October 2020, using nasopharyngeal swabs samples obtained from patients diagnosed with COVID-19 at our reference hospital, HB.

From October 3, 2020, to June 29, 2021, a total of 305 SARS-CoV-2 complete genomes were generated at the Laboratório de Pesquisas em Virologia (LPV), part of the Corona-ômica network (http://www.corona-omica.br-mcti.lncc.br), at Faculdade de Medicina de São José do Rio Preto (FAMERP), from samples obtained in SJdRP (88.8% of the sequences) and 15 neighboring municipalities (10.2% of the sequenced genomes) (Supplementary Data 1). Samples were obtained from 163 female (53.4%) and 142 male patients (46.5%), with an average age of 43 years (age range of 8-months-old to 91-years-old) (Supplementary Fig. 2).

### Lineage classification of SARS-CoV-2 genomes

**Fig. 2:**
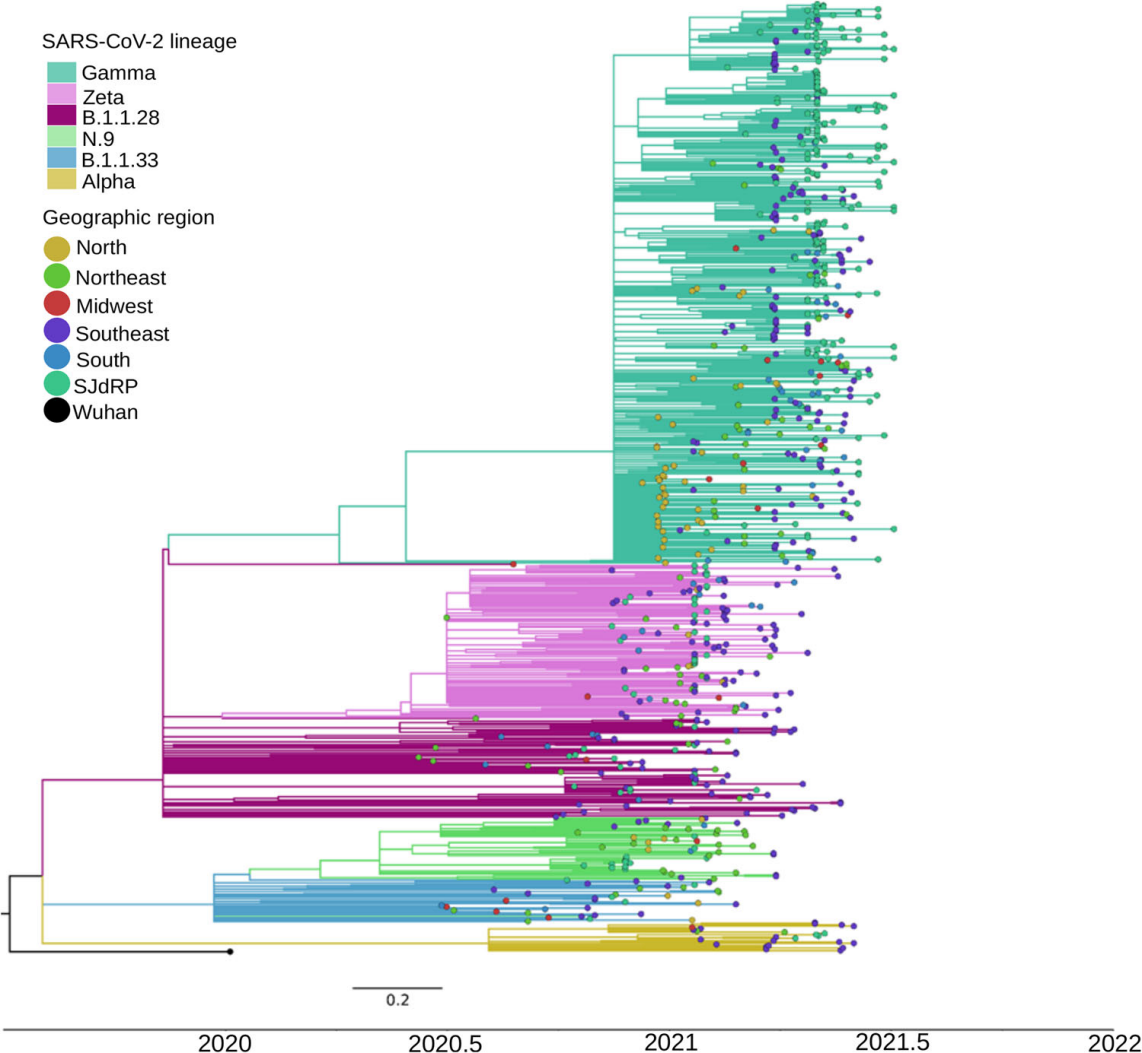
Maximum likelihood tree of SARS-CoV-2 based on complete genome sequences from São José do Rio Preto and all Brazilian regions. Phylogenetic tree reconstructed using GTR + F + R2 as nucleotide substitution model. The reliability of branching patterns was tested using Ultrafast Bootstrap (UFBoot) combined with SH-like Approximate Likelihood-ratio test (SH-aLRT). The analysis involved 272 complete genome sequences from SJdRP and 509 from five Brazilian regions. The analysis was conducted in IQ-TREE v. 2.0.3, and the final tree was visualized and edited in FigTree v.1.4.4. Branches are colored according to SARS-CoV-2 lineages classified by Pangolin v.3.1.14. Tip nodes are colored according to the origin of the sequences (except the reference sequence from Wuhan), which are from the study area of São José do Rio Preto (SJdRP) and all the Brazilian regions (North, Northeast, Midwest, South, and Southeast). Branch lengths are represented by a scale bar (labelled 0.2) at the bottom of the phylogenetic tree.

SARS-CoV-2 viral lineages (sequences obtained in this study and available at GISAID database) were classified using Pangolin version 3.1.14^2^ showing that the most prevalent lineages circulating in SJdRP were classified into B.1.1.28 (*n* = 15, 5.5%), N.9 (*n* = 14, 5.1%), Zeta (*n* = 28, 10.3%), Gamma (*n* = 194, 71.5%) and others (which includes B.1.1, B.1.2, B.1.1.332, B.1.1.33, B.1.177, Alpha and P.1.1, P.1.2, P.1.7 (*n* = 20, 7.3%)) (Supplementary Data 2 and 3). The genome sequencing coverage nine months of the pandemic, corresponding to period with the most number of COVID-19 confirmed cases (Supplementary Fig. 3). The variant prevalence changed over time, from six lineages co-circulating in October, November, and December 2020, with a predominance of N.9 (34.3%) and B.1.1.28 (28.1%), to seven lineages in January and February 2021. Additionally, a shift in the prevalence of variants was observed, represented by a fourfold decrease in N.9 frequency (7.9% of sequenced genomes) and an increase in Zeta variant frequency (63.1% of sequenced genomes). Moreover, in January 2021, Gamma lineage was first detected and contributed to changing the landscape of circulating variants in SJdRP (Fig. 1d) and surrounding cities (Supplementary Fig. 4 and Supplementary Data 4 and 5) for the subsequent months. Critically, following its introduction, it displayed a rapid increase in prevalence, reaching more than 95% of the sequenced genomes from March to June, thus replacing Zeta and other lineages (Fig. 2). In April 2021, the VOC Alpha was first detected in SJdRP, and despite its low frequency, it was the only variant identified in May and June, together with Gamma variants (P.1, P.1.1, and P.1.2) (Fig. 1d). Similarly, a complete replacement of other variants by Gamma was also observed in neighboring municipalities of SJdRP, administered by the Regional Health Department XV (RHD XV), for which SJdRP serves as the main health center (Supplementary Fig. 4).

### SARS-CoV-2 Gamma variant introduction and clade replacement

We performed a maximum likelihood phylogeographic analysis using a dataset composed of 781 Brazilian complete genomic sequences of B.1.1.28, Gamma, Zeta, N.9, B.1.1.33, and Alpha variants, from which 262 were obtained in this study and ten are sequences from SJdRP retrieved from the GISAID database (Supplementary Data 6). Our genomic surveillance indicates that multiple introduction events of different SARS-CoV-2 lineages occurred in SJdRP from several Brazilian regions (Fig. 2). The B.1.1.28 variant (*n* = 16), the ancestral lineage of Gamma and Zeta, was introduced in SJdRP several times over at least five months. The most prevalent lineages, Gamma (*n* = 200) and Zeta (*n* = 31), formed a well-supported group (UFBoot = 100 and 100, SH-aLRT = 99.8 and 92.5, respectively), including sequences from four Brazilian geographic regions, further supporting the notion of repeated strain introductions into the area (Fig. 2).

**Fig. 3:**
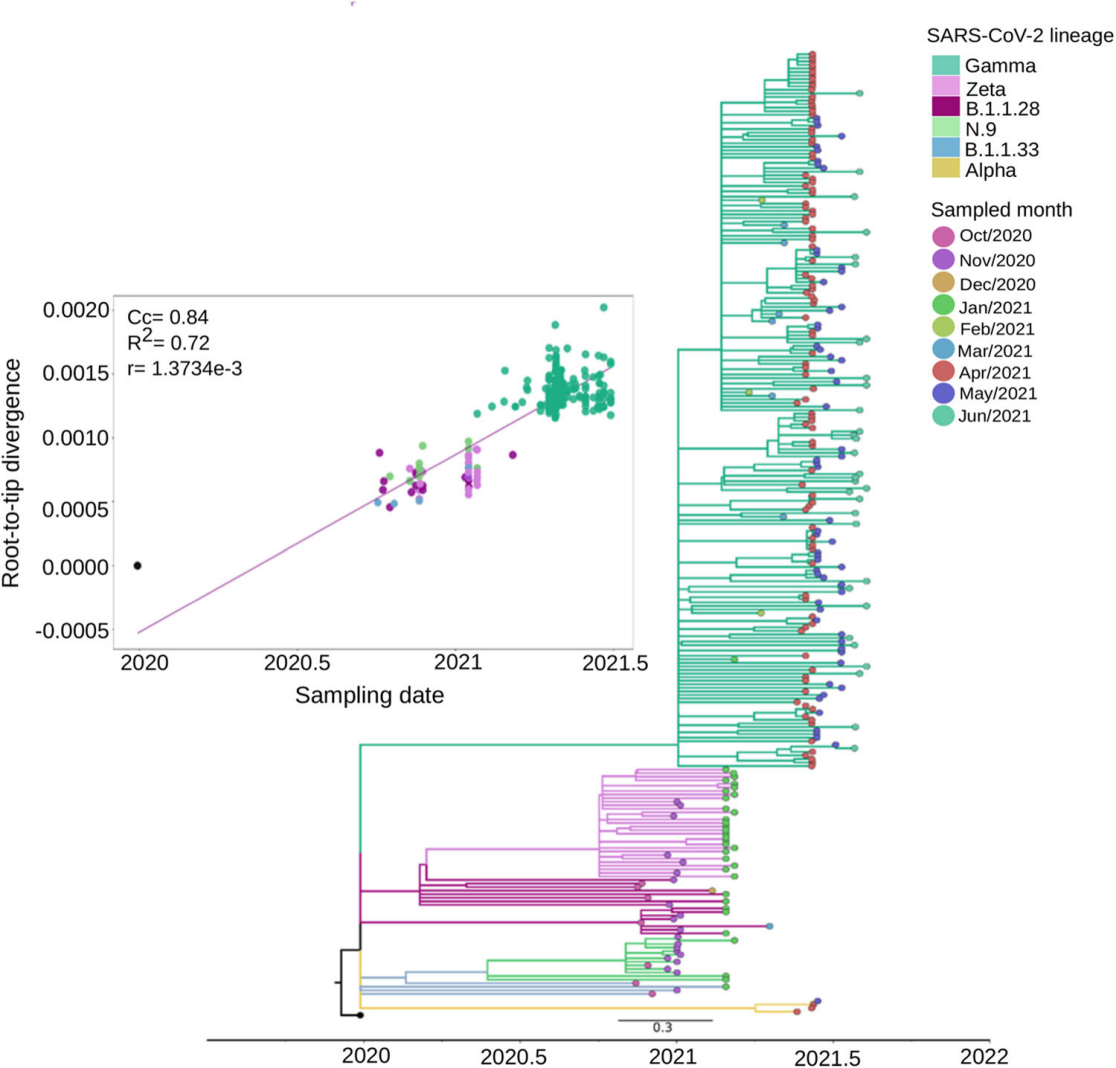
Maximum likelihood tree of SARS-CoV-2 based on complete genome sequences from São José do Rio Preto, from October 2020 to June 2021, according to sampled month. The Maximum-Likelihood tree was reconstructed based on the GTR + F + R3 nucleotide substitution model. The reliability of branching patterns was tested using the Ultrafast Bootstrap (UFBoot) combined with SH-like Approximate Likelihood-ratio test (SH-aLRT). The analysis involved 272 complete genome sequences. The analysis was conducted in IQ-TREE v. 2.0.3, and the final tree was visualized and edited in FigTree v.1.4.4. Correlation between the sampling date of the most prevalent SARS-CoV-2 lineages detected in SJdRP and their genetic distance from the root (hCoV-19/Wuhan/WIV04/2019 - EPI_ISL_402124) based on the Maximum Likelihood phylogenetic tree (Correlation coefficient (Cc) = 0.84; *R*2 = 0.72, substitution rate (*r*) = 1.3734e-3). Branches are colored according to SARS-CoV-2 lineage classified by Pangolin v. 3.1.14. Tip nodes are colored (except the reference sequence from Wuhan) according to the sampled month. Branch lengths are represented by a scale bar (labelled 0.3) at the bottom of the phylogenetic tree.

Due to a large number of SARS-CoV-2 Gamma sequences detected in our genomic surveillance, we performed a more targeted phylogenetic analysis of all sequences sampled solely from SJdRP and the surrounding area (Supplementary Data 7 and 8). Gamma sequences formed a single well-supported group (UFBoot = 100, SH-aLRT = 100) clustered most closely with B.1.1.28 sequences, the parental lineage of Gamma (Fig. 3). The first detection of Gamma lineage in SJdRP was on 26th January 2021, followed by a rapid increase in its prevalence over other circulating variants in less than two months (Fig. 3) (January to March 2021). Root-to-tip analyses confirmed that Gamma was the most divergent variant (Fig. 3). Interestingly, Gamma samples collected at the same period displayed different divergence patterns, a diversification process in the Gamma variant circulating in SJdRP, supported by the large number of reported cases observed (Fig. 3).

### Epidemiologic profile of the introduction of SARS-CoV-2 Gamma variant

**Fig. 4:**
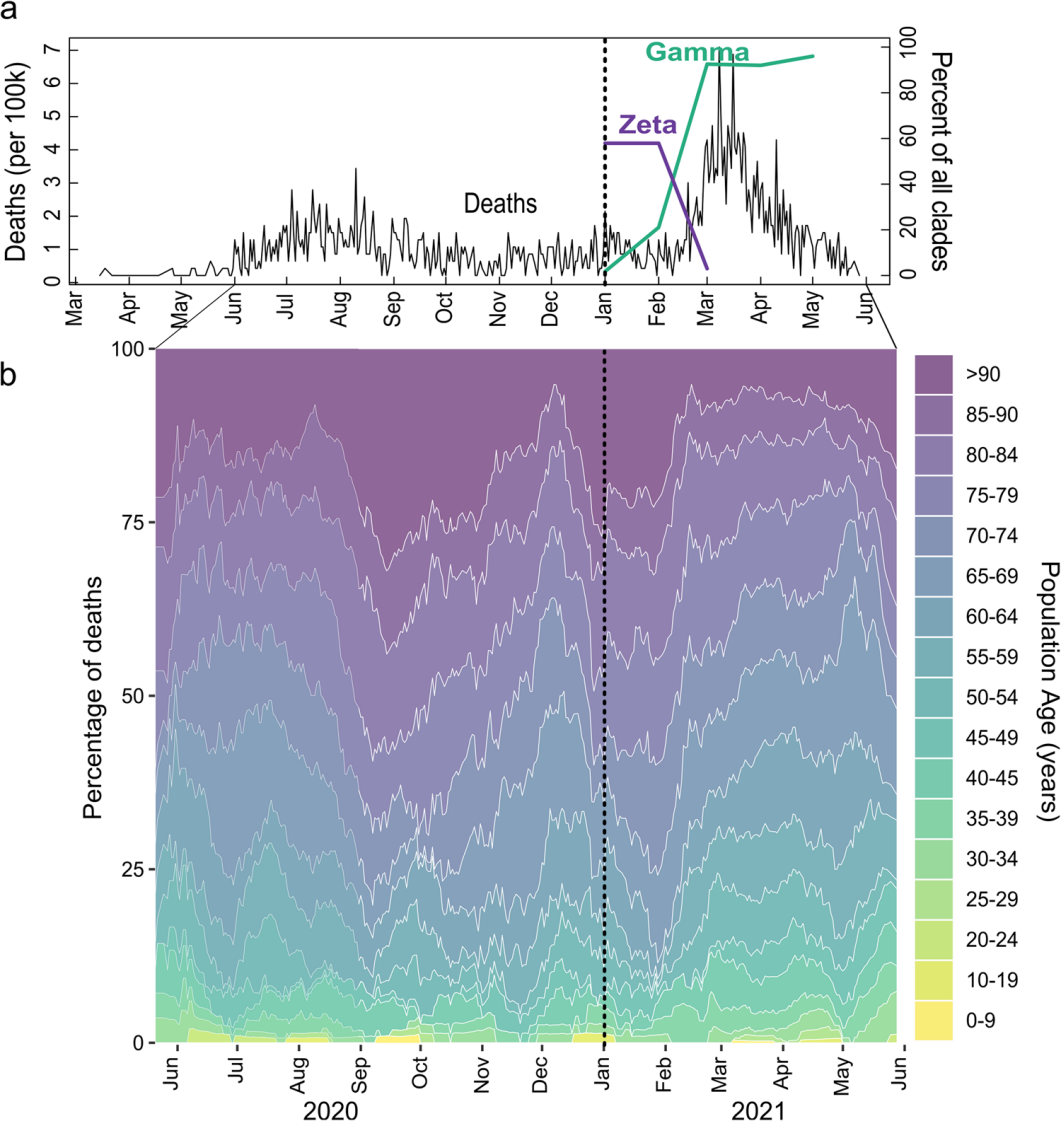
COVID-19 deaths from March 2020 to June 2021 showing an increase associated with SARS-CoV-2 Gamma lineage introduction and spread in São José do Rio Preto, Brazil. **a** COVID-19 deaths per 100,000 inhabitants (black line) from March 2020 to June 2021 (left *y*-axis) and proportion of Gamma (green line) and Zeta (lilac line) lineages detected (right *y*-axis). **b** Percentage of COVID-19 deaths by population age (years). Gamma: SARS-CoV-2 Pangolin lineage P.1. Zeta: SARS-CoV-2 Pangolin lineage P.2.

Deaths showed a marked increase as variant Gamma became dominant (Fig. 4a), increasing by 166% (95% CI: 127, 214, *p* < 0.001) when comparing July to September 2020 to March to April 2021. While the over 70 years of age group accounted for most deaths across the epidemic, shifts were seen from older to younger age groups as Zeta waned and Gamma dominated (Fig. 4b).

**Fig. 5:**
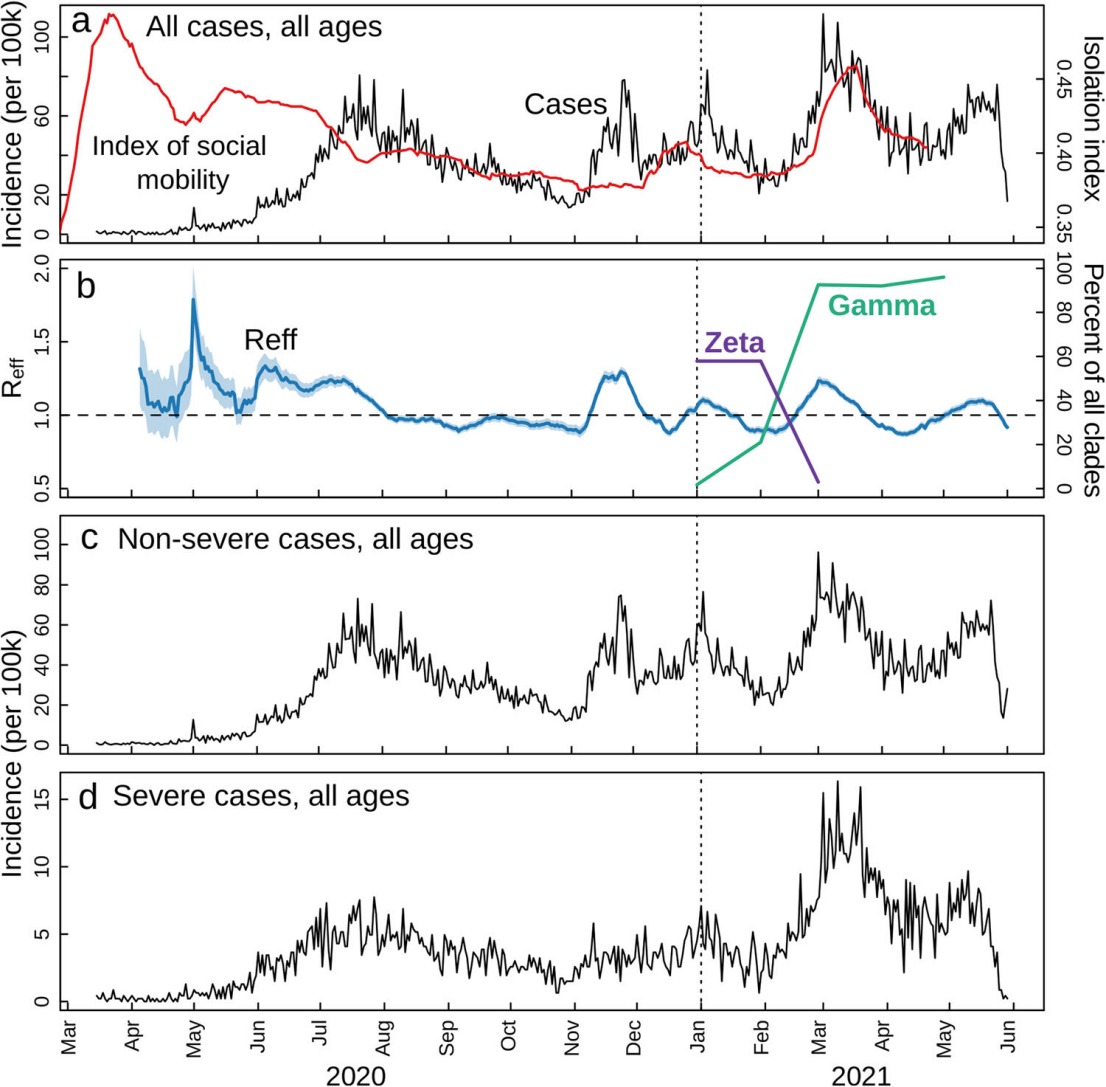
COVID-19 incidence per 100,000 inhabitants from March 2020 to June 2021 in São José do Rio Preto, Brazil. **a** COVID-19 incidence per 100,000 inhabitants for total cases. Index of social mobility: measure based on individual mobility reports. **b** Estimates of effective reproductive number (Reff) overtime (blue line) for all COVID-19 cases (left *y*-axis), as well as the percentage of Gamma (green line) and Zeta (lilac line) (right *y*-axis). Error bars indicate 95% confidence intervals for Reff estimates, *n* = 75,323 cases for calculation. **c** COVID-19 incidence per 100,000 inhabitants for non-severe cases. **d** COVID-19 incidence per 100,000 inhabitants for severe cases.

**Fig. 6:**
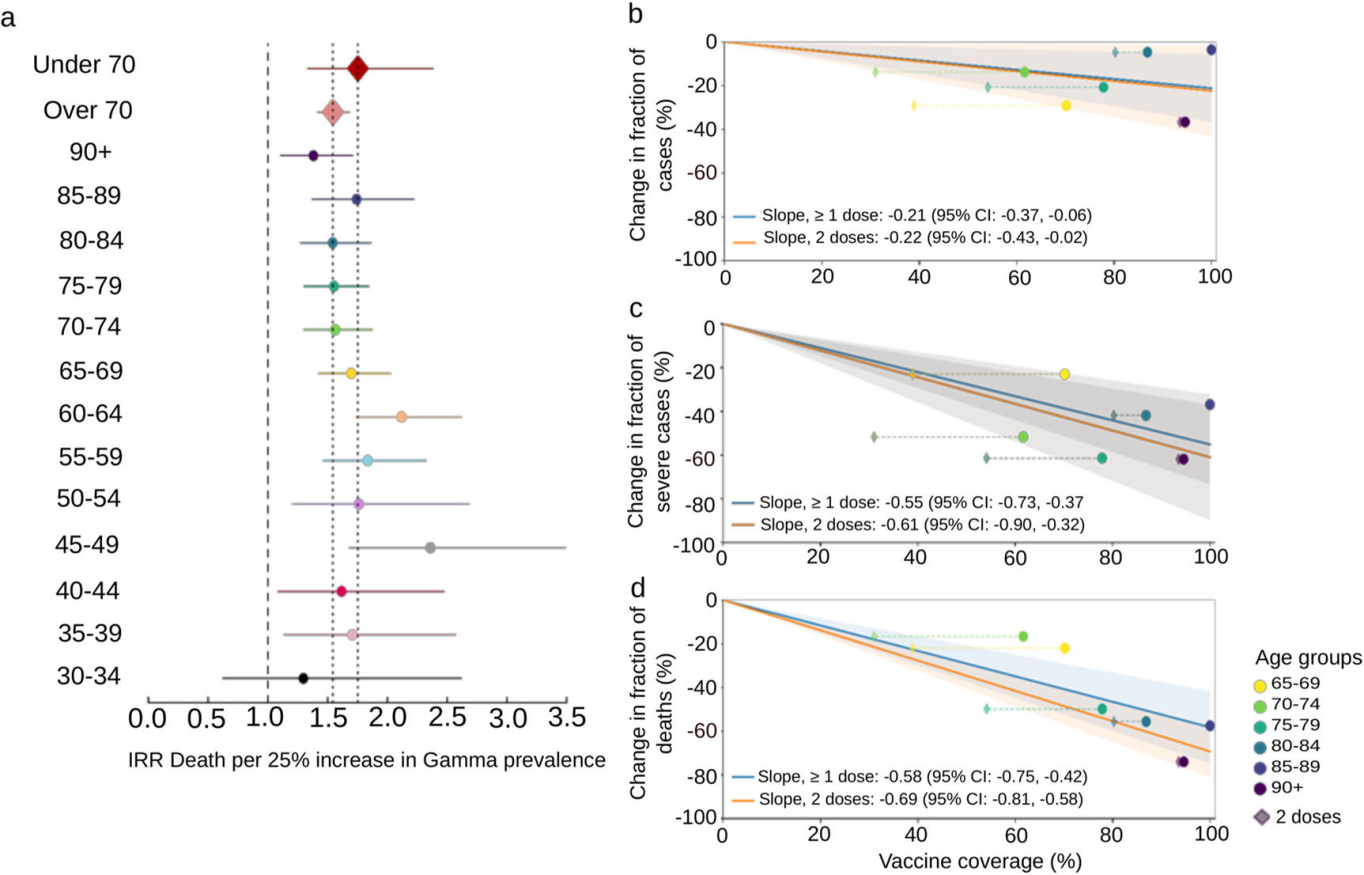
Increased COVID-19 mortality risk associated with SARS-CoV-2 Gamma lineage introduction and estimated impact of COVID-19 vaccination on transmission, severity, and mortality in São José do Rio Preto, Brazil. **a** Incidence rate ratio (IRR) for death for each 25% increase in Gamma prevalence, adjusted for per-day numbers of tests performed. Error bars indicate 95% confidence intervals. Diamonds of different colors represent the IRR death per 25% increase in Gamma prevalence for all individuals under 70 (*n* = 962) or over 70 years (*n* = 1210). Data of deaths by each age group and per day is available on Github repository^49^. The circles of different colors represent the IRR death per 25% increase in Gamma prevalence for each age group. **b** Change in the proportion of cases in each age group from before versus after vaccination. Circles show effective vaccine coverage (total doses divided by twice the population), while diamonds show the percentage of people who have received both doses, along with the line of best fit and 95% confidence interval (shaded area) for each. **c**, **d** Change in proportions of severe cases and deaths, respectively.

Cases per 100,000 fluctuated from March 2020 to June 2021, with corresponding estimates of the effective reproduction number (Reff) (Fig. 5a, b). The Reff reflects the behavior of an epidemic, which is strongly related to an index of social mobility of a population. The Reff is the average number of secondary infections caused by an infected person, where *R* > 1 indicates a growing epidemic, while an *R* < 1 indicates a decrease in transmission^51^. Peaks in cases and Reff were seen in July to August 2020, November 2020, January 2021, and the highest peaks in March and April 2021, followed by a reduced index of social mobility (Fig. 4a) and the rise in dominance of Gamma from March 2021. Non-severe cases were nearly 10-fold higher than severe cases during most of the time of the pandemic; however, an increase of 127% (95% CI: 105, 152, *p* < 0.001) of severe cases was seen in March 2021, corresponding to the rise of Gamma (Fig. 5c, d). Proportionally, the largest increase in severe cases was seen in those under 70 years old (109% increase comparing January to March 2021; 95% CI: 78, 149, *p* < 0.001) as opposed to those over 70, which had a 19% (95% CI: 7, 33, *p* < 0.001) increase (Supplementary Fig. 5). Negative binomial regressions were far superior by AIC than Poisson models. Incidence rate ratios (IRRs) for death ranged from 1.3 (95% CI: 0.63, 2.62, *p* = 0.37) for those aged 30–34 to 2.36 (95% CI: 1.68, 3.49, *p* < 0.001) for those aged 45–49 (Fig. 6a). Correspondingly, IRRs were higher overall for those under 70 versus over 70 (1.75, 95% CI: 1.34, 2.38, *p* < 0.001 versus 1.54, 95% CI: 1.42, 1.68, *p* < 0.001), indicating that people aged from 45 to 69 present higher mortality rate due to Gamma infection, likely due to less vaccination coverage by the time of the study (Fig. 6a). Additionally, daily surveillance of the available Intensive Care Unit (I.C.U.) beds for COVID-19 patients in SJdRP revealed that the occupancy rate reached 100% only in a few days in March 2021, corresponding to the increase of Gamma prevalence and the number of severe cases (Supplementary Fig. 6).

### Effects of COVID-19 vaccination

CoronaVac and AstraZeneca vaccines were rolled out to people in descending age order, beginning with those over 90 years old on 2 February 2021. To estimate the impact of vaccination, we compared the proportion of cases, severe cases, and deaths in each age group before the rollout (for that age group) versus two weeks after the rollout onwards. A line with a fixed intercept was fit to these changes in proportion, such that a slope of –1 corresponds to one case, severe case, or death being averted per person vaccinated. Vaccination was associated with a moderate reduction in the number of cases (best-fit slope –0.21, 95% CI: –0.03, –0.39) (Fig. 6b). However, it was associated with a pronounced reduction in severe cases (–0.55, 95% CI: –0.34, –0.76) and deaths (–0.58, 95% CI: –0.39, –0.77) (Fig. 6c, d). A slope of less than –1 for deaths, for example, would imply that more than one death is averted per person vaccinated; this would be the case if, for example, people who are vaccinated not only have significantly lower mortality rates if infected but if they also have lower rates of transmission, which preferentially reduces infection rates among unvaccinated people in the same age group (herd immunity).

## Discussion

For more than eighteen months, the world has lived amid a pandemic, with an alarming number of cases and deaths and devastating consequences in all sectors of society. Nevertheless, the current situation in some countries is encouraging, with the number of cases and deaths rapidly decreasing following mass vaccination. Unfortunately, Brazil remains an epicenter of SARS-CoV-2 transmission due to the persistent absence of control measures and low vaccination rates^52^. From the first recorded case in February 2020 to November 2021, Brazil recorded more than 21.8 million laboratory-confirmed cases with more than 600 thousand deaths^52^. Using a combination of whole-genome sequencing and epidemiological analysis, we show that the introduction, rapid spread, and dominance of SARS-CoV-2 Gamma lineage in SJdRP was associated with an increase of severe COVID-19 cases and deaths. Moreover, the mortality risk related to Gamma infection largely depends on several factors, including age, underlying conditions, and lack of vaccination.

The Gamma lineage was first detected in early December in Manaus, the capital of Amazonas state, and it quickly spread to other Brazilian states, mainly to the South-eastern region^13^. In early February 2021, a total of 41 complete genomes of SARS-CoV-2 Gamma lineage were available in the GISAID database (4.8% from Pará, 12.1% from São Paulo, and 82.9% from Amazonas states). More than 49 thousand Gamma genomes have been deposited worldwide, of which 27.14% were contributed from Brazil^53^. Our genomic surveillance documented the first detection of Gamma lineage in SJdRP on January 26, 2021, only one month after its detection in Manaus. Following its introduction in the region, this variant quickly became the most dominant lineage, outplacing eight circulating lineages of SARS-CoV-2. Despite the low number of sequenced genomes compared with the total of confirmed cases, one limitation of our study, we were able to detect nine circulating variants from October 2020 to January 2021 (including the first detection of Gamma lineage). This result reflects a several number of circulating variants, even considering a small number of sampled genomes (*n* = 68). After the Gamma introduction in the city and the higher number of cases observed, we have increased our genomic surveillance, and in the period from February to June, a total of 203 genomes from SJdRP were sequenced, from which 193 (95.0%) were classified as Gamma variant, showing a clear clade replacement event. These observations mirror trends reported in Manaus and other Brazilian regions^13,54,55^, attributing its rapid spread and dominance to its higher transmissibility rate and greater viral fitness^13^. This finding is concerning as clade replacement can maintain disease in populations through successive introductions of new lineages^56^, especially in countries considered epicenters of the pandemic, as shown previously with dengue and Influenza viruses^57–62^.

We demonstrate that a clade replacement event driven by the introduction of Gamma lineage was able to change the epidemiological landscape of COVID-19 in the municipality. The detection and prevalence of Gamma lineage led to a prominent peak in the total cases and deaths recorded in SJdRP. It is important to point out that despite the first wave of SARS-CoV-2 infections in Brazil may have a role in decreasing disease severity, by conferring herd immunity to the population, the emergence of Gamma lineage was crucial to increase the number of severe cases and deaths by COVID-19 in early 2021, as observed in our study. This result indicates that prior infection may not confer long-lasting immunity^63^, mainly against variants that present higher transmissibility and immune evasion^13^, reinforcing that massive vaccination is essential to attenuate the number of cases and deaths. Confirming this fact, Buss and colleagues showed^63^ that in October 2020, more than 70% of the population from Manaus was infected by SARS-CoV-2, which was not able to prevent a rapid increase in deaths and cases in the second epidemic wave, which began at the end of December 2020^13,64^.

Interestingly, our results demonstrate a 109% increase in the number of COVID-19 severe cases of individuals under 70 years after Gamma detection in SJdRP, while in the group of individuals over 70 years, there was only a 19% increase of severe COVID-19 cases. This finding highlights the impact of recent vaccinations on older individuals, resulting in remarkable reductions in severe disease and mortality. Our findings of the incidence rate ratio of deaths by COVID-19 infection after Gamma introduction revealed a higher mortality risk in people under 70 years, which was more accentuated in individuals aged 45–64 years old. These results are like other recent work on the Gamma variant, which reported notable increases in the case fatality rate in those under 50 years of age after the introduction of Gamma in the Brazilian state of Paraná (state in the south of São Paulo state)^55^.

Furthermore, our results highlight the urgency of rapid and thorough vaccination in all age groups. We showed a pronounced reduction in severe cases and deaths in age groups that have received either one or both doses of SARS-CoV-2 vaccines. Studies have reported that besides the protective effect of vaccines against SARS-CoV-2 variants, mainly in severe cases^30,31,33,34,65,66^, vaccination can reduce the viral load in infections occurring 12–37 days after the first dose^67^, therefore suppressing virus spread. Thus, if efficient vaccination measures are taken rapidly, it is possible to lower the transmission rate and the mortality risk for all age groups. Moreover, a rapid and efficient vaccination program is essential to reducing virus divergence, and thus, the emergence of new and more concerning SARS-CoV-2 variants can drive new clade replacement events.

### Reporting summary

Further information on research design is available in the Nature Research Reporting Summary linked to this article.

## Supplementary information

Supplementary Information

Supplementary Data 1

Supplementary Data 2

Supplementary Data 3

Supplementary Data 4

Supplementary Data 5

Supplementary Data 6

Supplementary Data 7

Supplementary Data 8

Description of Additional Supplementary Files

Reporting Summary

## Data Availability

All source data used to generate the maps are available at Supplementary Data 1–6. All SARS-CoV-2 genomes generated and analyzed in this study are available at the EpiCoV database in GISAID, and their respective access numbers are available at Supplementary Data 1, 2, 4, 6, 7, and 8. All data used to perform epidemiological analyses and graphs are stored in our publicly available Github repository (https://github.com/amath-idm/brazil_p1_replacement)^50^. The mobility data used in this study is available at https://www.saopaulo.sp.gov.br/coronavirus/isolamento/.

## Code availability

Codes for the epidemiologic analyses and vaccination efficacy are available on GitHub (https://github.com/amath-idm/brazil_p1_replacement)^50^. The version of the code described in the paper is deposited in the DOI-minting repository Zenodo^50^.
