# Supplementary material for: Impact of SARS-CoV-2 Gamma lineage introduction and COVID-19 vaccination on the epidemiological landscape of a Brazilian city
Source: Commun Med (Lond). 2022 Apr 13;2:41. doi: 10.1038/s43856-022-00108-5 (PMC9053258; doi:10.1038/s43856-022-00108-5)
Supplement: Supplementary file 1 — Supplementary Information [file 43856_2022_108_MOESM1_ESM.pdf]

Impact of SARS-CoV-2 Gamma lineage introduction and COVID-19 vaccination on the epidemiological landscape of a Brazilian city

Cecília Artico Banho[1#], Lívia Sacchetto[1#], Guilherme Rodrigues Fernandes Campos[1], Cíntia Bittar[2], Fábio Sossai Possebon[3], Leila Sabrina Ullmann[3], Beatriz de Carvalho Marques[1], Gislaine Ceslestino Dutra da Silva[1], Marília Mazzi Moraes[1], Maisa Carla Pereira Parra[1], Andreia Francesli Negri[4], Ana Carolina Boldrin[4], Michela Dias Barcelos[4], Thayza M. I. L. dos Santos[1], Bruno H. G. A. Milhim[1], Leonardo Cecílio Rocha[1], Fernanda Simões Dourado[1], Andresa Lopes dos Santos[1], Victoria Bernardi Ciconi[1], Caio Patuto[1], Alice Freitas Versiani[1], Rafael Alves da Silva[1], Edoardo Estevam de Oliveira Lobl[1], Victor Miranda Hernandes[1], Nathalia Zini[1], Carolina Colombelli Pacca[1,5], Cássia Fernanda Estofolete[1], Helena Lage Ferreira[6], Paula Rahal[2], João Pessoa Araújo Jr.[3], Jamie A. Cohen[7], Cliff C. Kerr[7], Benjamin M. Althouse[7,8,9], Nikos Vasilakis[10,11,12,13,14], Mauricio Lacerda Nogueira[1,10*]

Supplementary Information

Supplementary Figure 1: Moving average of daily cases from March 2020 to May 2021.

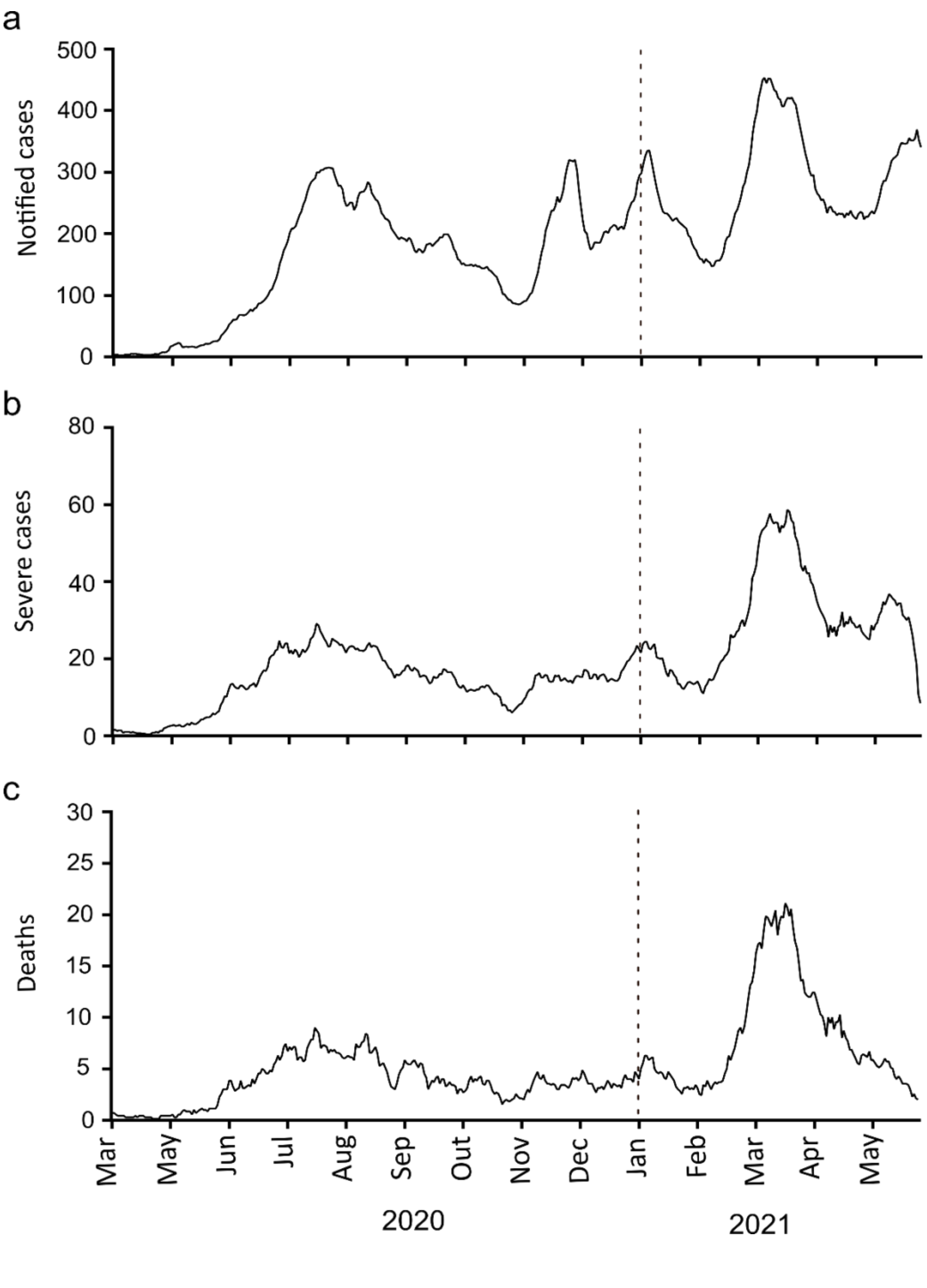

a, Moving average of total notified cases. **b,** Moving average of severe cases. **c,** Moving average of deaths in the Regional Health Department XV (RHD XV) of São José do Rio Preto.

Supplementary Figure 2: Sociodemographic data of the studied population.

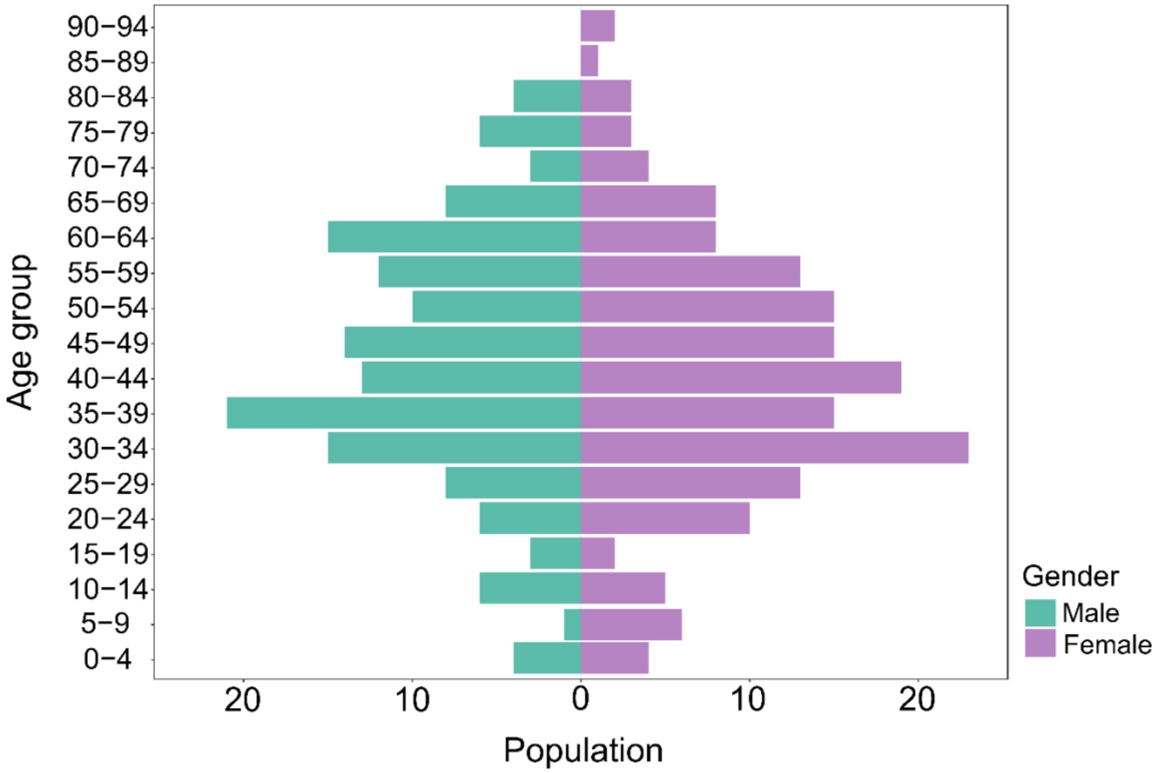

Sociodemographic data from 318 samples, from which SARS-CoV-2 complete genomes were generated. The samples were obtained from 169 female and 149 males patients, which presented an average age of 43 years old.

Supplementary Figure 3: Genome sequencing coverage from October 2020 to June 2021.

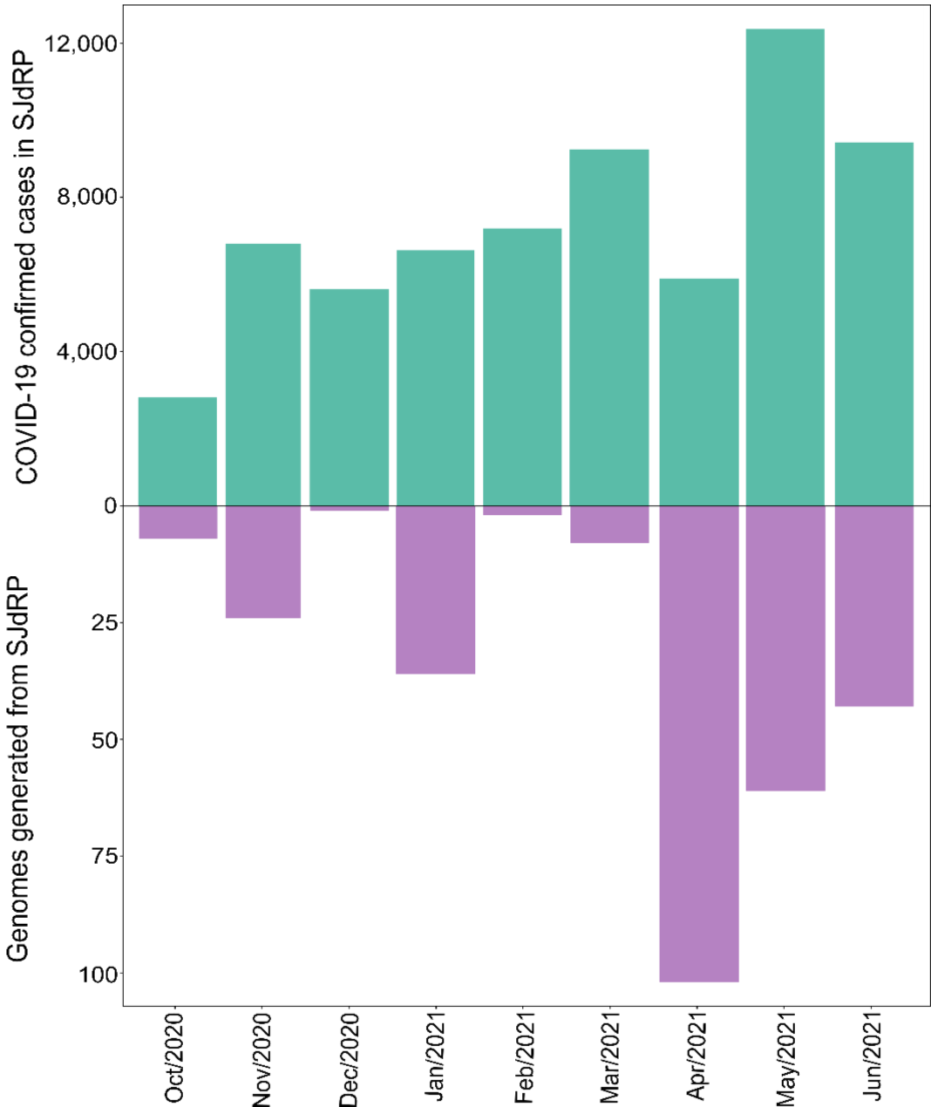

Number of confirmed cases for COVID-19 in SJdRP from October 2020 to June 2021 and the number of sequenced genomes for the period.

Supplementary Figure 4: Geographic and temporal distribution of SARS-CoV-2 variants detected in the Regional Health Department (RHD XV) of São José do Rio Preto, from October 2020 to June 2021 by genomic surveillance.

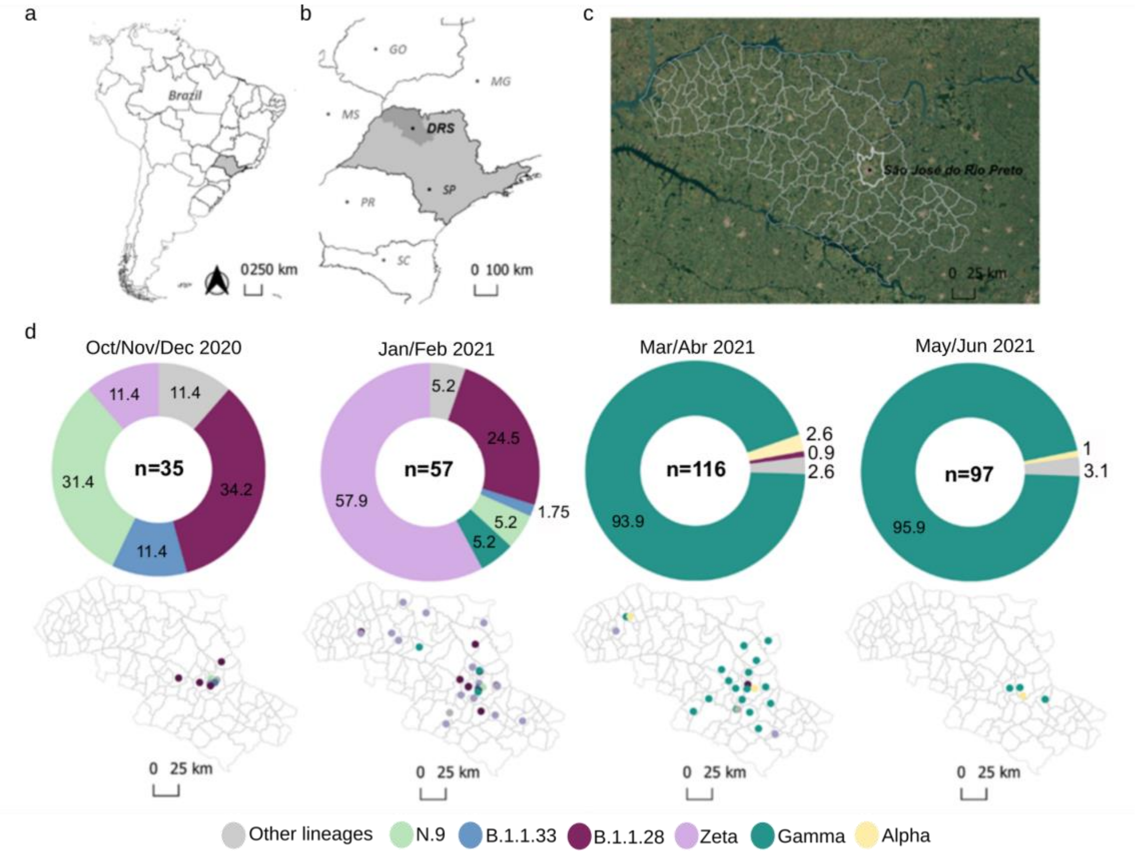

a, Geopolitical map of Brazil and São Paulo state highlighted in grey. **b,** Map of São Paulo state (in grey) indicating the Regional Health Department (RHD XV) of São José do Rio Preto (SJdRP), located in the Northwest region. **c,** Satellite image of RHD XV generated using the Google Maps Geocoder v.0.0.3 plugin implemented in QGIS software version 3.8.2 (http://qgis.org). **d,** Prevalence, and distribution of SARS-CoV-2 variants detected the RHD XV of SJdRP from October 2020 to June 2021 by genomic surveillance. The information about the detected SARS-CoV-2 variant's location and collection date was used to create the maps in QGIS software version 3.8.2 (http://qgis.org).

Supplementary Figure 5: Increase of case severity associated with SARS-CoV-2 Gamma lineage introduction and spread in São José do Rio Preto.

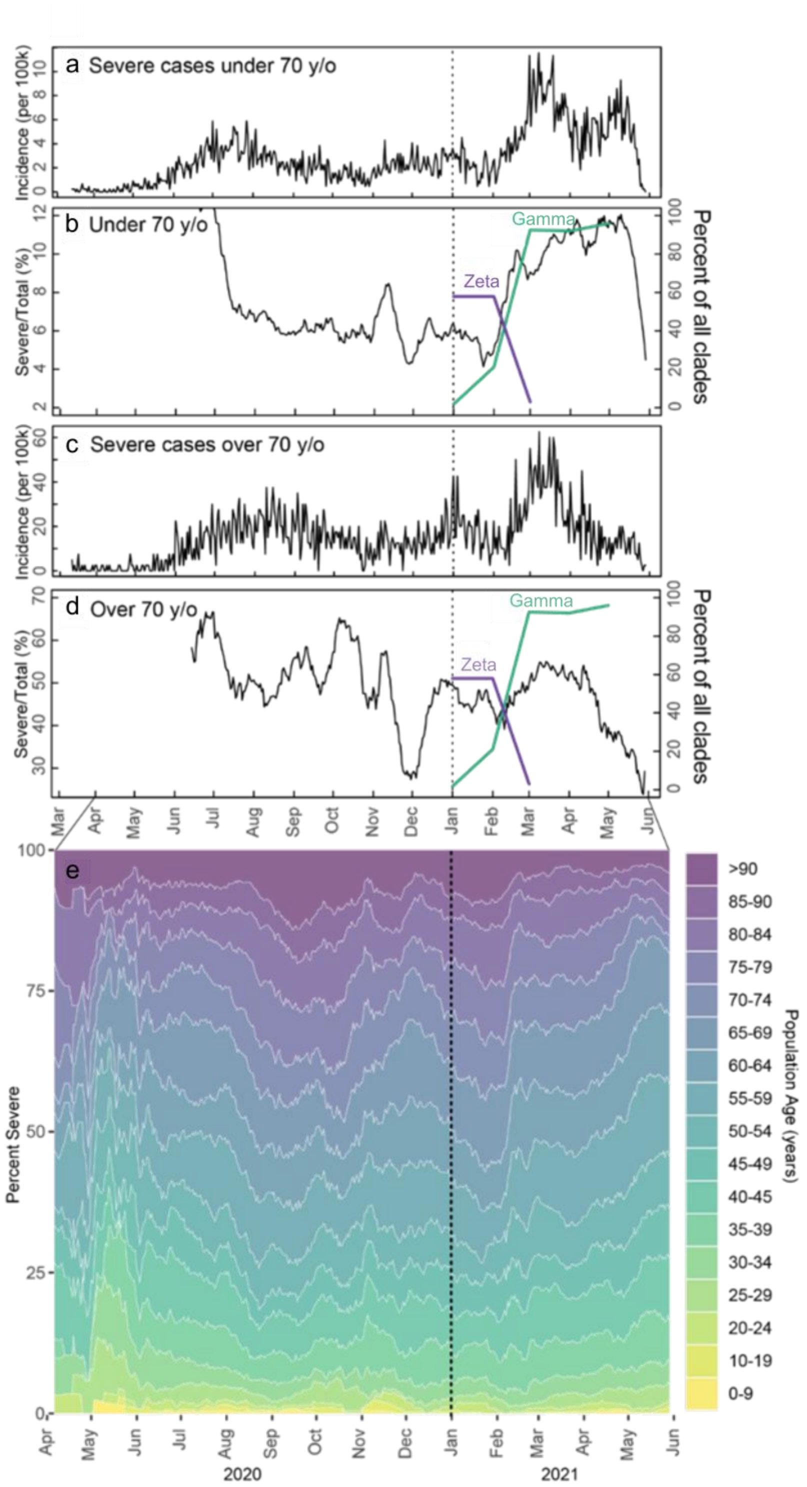

a, b, c, d, incidence of SARS-CoV-2 and the proportion of COVID-19 cases that are severe for those under 70 and over 70 years, respectively. **e,** age breakdown of severe cases by age and time.

Supplementary Figure 6: Percentage of Intensive Care Unit (I.C.U.) beds occupied per month.

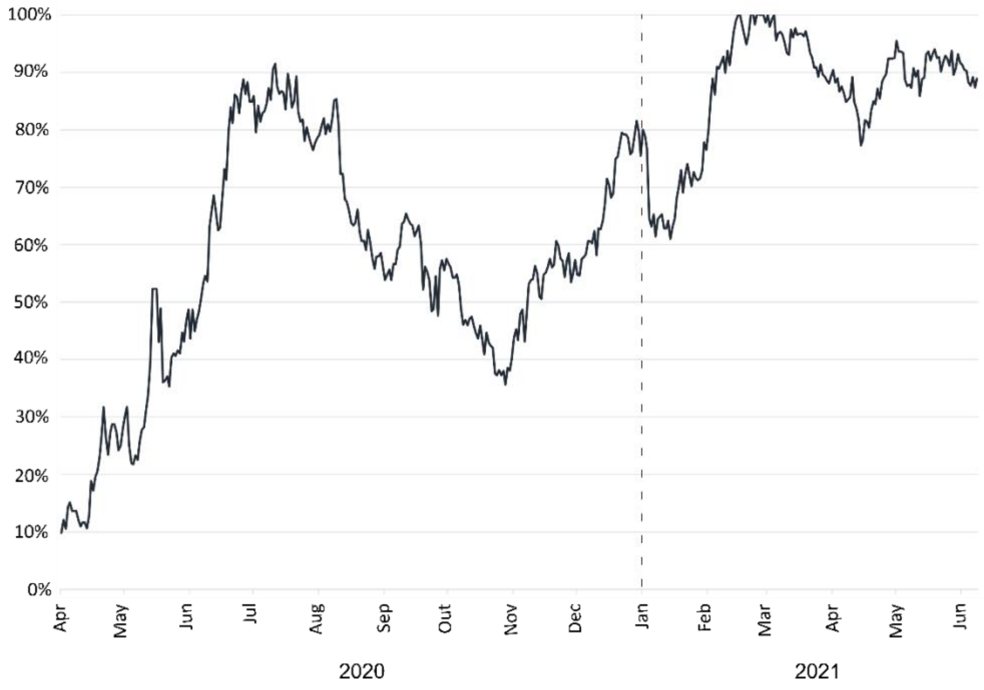

Rate of Intensive Care Unit (I.C.U.) beds occupied by patients diagnosed with COVID-19 in São José do Rio Preto.
