# Supplementary material for: Impact of SARS-CoV-2 Gamma lineage introduction and COVID-19 vaccination on the epidemiological landscape of a Brazilian city
Source: Commun Med (Lond). 2022 Apr 13;2:41. doi: 10.1038/s43856-022-00108-5 (PMC9053258; doi:10.1038/s43856-022-00108-5)
Supplement: Supplementary file 10 — Description of Additional Supplementary Files [file 43856_2022_108_MOESM10_ESM.pdf]

Description of Additional Supplementary Files

File Name: Supplementary Data 1

Description: SARS-CoV-2 whole genome sequences generated in this study, from October 2020 to June 2021.

All SARS-CoV-2 genomes generated and analyzed in this study are available at the EpiCoV database in GISAID (https://www.gisaid.org). FAMERP: Faculdade de Medicina de São José do Rio Preto. SJRP: São José do Rio Preto. ID: Identification.

File Name: Supplementary Data 2

Description: SARS-CoV-2 whole genome sequences generated in this study and retrieved from EpiCoV database in GISAID, from São José do Rio Preto.

All SARS-CoV-2 genomes generated and analyzed in this study are available at the EpiCoV database in GISAID (https://www.gisaid.org). ID: Identification.

File Name: Supplementary Data 3

Description: Prevalence of SARS-CoV-2 variants detected in SJdRP from October 2020 to June 2021 by our genomic surveillance

SJdRP: São José do Rio Preto. Oct.: October, Nov.: November, Dec.: December, Jan.: January, Feb.: February, Mar.: March, Apr.: April, Jun.: June.

File Name: Supplementary Data 4

Description: SARS-CoV-2 whole genome sequences generated in this study and retrieved from EpiCoV database in GISAID, from the Regional Health Department XV (RHD XV).

All SARS-CoV-2 genomes generated and analyzed in this study are available at the EpiCoV database in GISAID (https://www.gisaid.org). FAMERP: Faculdade de Medicina de São José do Rio Preto. SJRP:São José do Rio Preto. ID: Identification.

File Name: Supplementary Data 5

Description: Prevalence of SARS-CoV-2 variants detected in the Regional Health Department XV (RHD XV), from October 2020 to June 2021 by our genomic surveillance.

Oct.: October, Nov.: November, Dec.: December, Jan.: January, Feb.: February, Mar.: March, Apr.: April, Jun.: June.

File Name: Supplementary Data 6

Description: Maximum-Likelihood dataset based on SARS-CoV-2 whole genome sequences from São José do Rio Preto and all Brazilian regions.

All SARS-CoV-2 genomes generated and analyzed in this study are available at the EpiCoV database in GISAID (https://www.gisaid.org). ID: Identification.

File Name: Supplementary Data 7

Description: Maximum-Likelihood dataset based on SARS-CoV-2 whole genome sequences from São José do Rio Preto.

All SARS-CoV-2 genomes generated and analyzed in this study are available at the EpiCoV database in GISAID (https://www.gisaid.org). ID: Identification.

File Name: Supplementary Data 8

Description: Maximum-Likelihood dataset based on SARS-CoV-2 whole genome sequences from the Regional Health Department XV (RHD XV).

All SARS-CoV-2 genomes generated and analyzed in this study are available at the EpiCoV database in GISAID (https://www.gisaid.org). ID: Identification
